# Xerotolerant *Cladosporium sphaerospermum* Are Predominant on Indoor Surfaces Compared to Other *Cladosporium* Species

**DOI:** 10.1371/journal.pone.0145415

**Published:** 2015-12-21

**Authors:** Frank J. J. Segers, Martin Meijer, Jos Houbraken, Robert A. Samson, Han A. B. Wösten, Jan Dijksterhuis

**Affiliations:** 1 Applied and Industrial Mycology, CBS-KNAW Fungal Biodiversity Centre, Uppsalalaan 8, 3584 CT, Utrecht, The Netherlands; 2 Microbiology, Utrecht University, Padualaan 8, 3584 CH, Utrecht, The Netherlands; Leibniz Institute DSMZ-German Collection of Microorganisms and Cell Cultures, GERMANY

## Abstract

Indoor fungi are a major cause of cosmetic and structural damage of buildings worldwide and prolonged exposure of these fungi poses a health risk. *Aspergillus*, *Penicillium* and *Cladosporium* species are the most predominant fungi in indoor environments. *Cladosporium* species predominate under ambient conditions. A total of 123 *Cladosporium* isolates originating from indoor air and indoor surfaces of archives, industrial factories, laboratories, and other buildings from four continents were identified by sequencing the internal transcribed spacer (ITS), and a part of the translation elongation factor 1α gene (*TEF*) and actin gene (*ACT*). Species from the *Cladosporium sphaerospermum* species complex were most predominant representing 44.7% of all isolates, while the *Cladosporium cladosporioides* and *Cladosporium herbarum* species complexes represented 33.3% and 22.0%, respectively. The contribution of the *C*. *sphaerospermum* species complex was 23.1% and 58.2% in the indoor air and isolates from indoor surfaces, respectively. Isolates from this species complex showed growth at lower water activity (≥ 0.82) when compared to species from the *C*. *cladosporioides* and *C*. *herbarum* species complexes (≥ 0.85). Together, these data indicate that xerotolerance provide the *C*. *sphaerospermum* species complex advantage in colonizing indoor surfaces. As a consequence, *C*. *sphaerospermum* are proposed to be the most predominant fungus at these locations under ambient conditions. Findings are discussed in relation to the specificity of allergy test, as the current species of *Cladosporium* used to develop these tests are not the predominant indoor species.

## Introduction

Indoor fungal growth represents a global problem. For instance, about 25% of dwellings of social housing in the European Union show fungal growth [1, 2]. This causes disfigurement of the building materials and poses a health threat for the occupants and particularly for asthmatic and allergic patients [3, 4]. Indoor fungal growth is strongly increased after incidents of water damage caused for instance by flooding or leakage [5–8].

In outdoor air samples, species belonging to the genera *Aspergillus*, *Penicillium* and *Cladosporium* are commonly occurring. These genera are also predominant in indoor environments, indicating a strong correlation in fungal presence between indoor and outdoor air [9–12]. *Penicillium chrysogenum* and *Aspergillus versicolor* are particularly abundant in the indoor environment after water damage, while *Cladosporium* species dominate under ambient conditions [7, 13–15]. *Cladosporium* are widespread fungi. In nature, they are often found on dead plant material and on plant surfaces and are therefore considered as phylloplane fungi [16–18]. They have also been isolated from hypersaline environments, hence *Cladosporium halotolerans* [19, 20], and from soil and rocks such as artic rock [21–24]. These fungi can also be isolated from man-made products such as paint, food, textiles, books, glass windows and wall paper [25].

The genus *Cladosporium* comprises three species complexes, namely *C*. *cladosporioides*, *C*. *herbarum* and *C*. *sphaerospermum*, representing 169species [25]. The *Cladosporium* species complexes can be distinguished based on morphology and DNA sequences of the internal transcribed spacer (ITS), translation elongation factor 1α (*TEF*) and actin (*ACT*) loci [19, 20, 25, 26]. It has been stated that *C*. *cladosporioides* is the most abundant fungus in outdoor air [13]. As the composition of indoor species reflects the composition of the outdoor species we would expect to find *C*. *cladosporioides*. However, pilot studies of indoor samples at our institute suggested that members of the *C*. *sphaerospermum* species complex were predominant in the indoor environment. This prompted us to study a larger number of isolates collected over 8 years from locations with fungal problems. We identified these isolates using the sequences of 69 species from Bensch et. al. [25]. These 69 species comprise 2 species from outside a species complex and 7, 21 and 39 species from the *C*, *sphaerospermum*, *C*. *herbarum* and *C*. *cladosporioides* species complexes, respectively. We here show that *C*. *sphaerospermum* is the most predominant *Cladosporium* species complex in the indoor environment in general and on indoor surfaces in particular. The latter may be explained by the higher xerotolerance of this species complex when compared to *C*. *cladosporioides* and *C*. *herbarum*. This is of interest as most allergy tests are focused on *Cladosporium herbarum*, which has not been identified during this study.

## Materials and Methods

### Isolation of organisms

Isolates of indoor *Cladosporium* species were taken from the research collection of the Applied and Industrial Mycology (DTO) group of the CBS Fungal Biodiversity Centre. The isolates were collected during a period of time from 2006 to 2013 on request from residents or owners who noticed indoor fungal problems. The isolates originated from indoor environments including archives, industrial factories, laboratories, and other buildings with or without water damage (Table 1). In these situations usually both swab and air samples were collected. Air samples were taken by using a MAS-100 (Merck) air sampler. This samples an amount of air depending on the size of the room ranging from 15 to 1000 liter. The air is sampled on 2% malt extract agar supplemented with penicillin and streptomycin (MEA p/s) (Oxoid) or on dichloran 18% glycerol agar (DG18) (Oxoid). Samples of indoor surfaces were taken by using a sterile cotton swab or sellotape [27] and were also grown on MEA p/s or DG18. Strains were stored at -80°C in 30% glycerol, 0.025% Tween 80 and 0.025% agar or 30% glycerol, 0.01% Tween 80, 5 mM *N*-(2-acetamido)-2-aminoethanesulfonic acid (ACES), pH 6.8.

**Table 1:** Isolates of *Cladosporium* species identified and used in this study.

Species complex	Identified species	DTO nr.	Isolation	CBS nr.		Geographical origin	ITS	*TEF*	*ACT*
*Cladosporioides*	*C*. *acalyphae*	086-C5	December-08		S	s Hertogenbosch, The Netherlands	KP701887	KP701764	KP702010
*Cladosporioides*	*C*. *angustisporum*	127-E6	April-09		A	USA	KP701935	KP701812	KP702057
*Cladosporioides*	*C*. *australiense*	072-C8	July-08	139572	A	Amsterdam, The Netherlands	KP701873	KP701750	KP701996
*Cladosporioides*	*C*. *australiense*	082-E3	November-08		A	Amsterdam, The Netherlands	KP701878	KP701755	KP702001
*Cladosporioides*	*C*. *australiense*	090-D2	January-09		S	Rijswijk, The Netherlands	KP701899	KP701776	KP702022
*Cladosporioides*	*C*. *australiense*	109-E8	October-09		S	Denmark	KP701914	KP701791	KP702037
*Cladosporioides*	*C*. *australiense*	255-F3	March-13		S	Amersfoort, The Netherlands	KP701978	KP701855	KP702100
*Cladosporioides*	*C*. *cladosporioides*	039-G6	April-07	112388	A	Germany	KP701868	KP701745	KP701991
*Cladosporioides*	*C*. *cladosporioides*	071-G1	July-08	139571	Indoor	Greece	KP701872	KP701749	KP701995
*Cladosporioides*	*C*. *cladosporioides*	082-F1	November-08		A	Weert, The Netherlands	KP701879	KP701756	KP702002
*Cladosporioides*	*C*. *cladosporioides*	090-C6	January-09		S	Rijswijk, The Netherlands	KP701898	KP701775	KP702021
*Cladosporioides*	*C*. *cladosporioides*	102-A4	May-09		S	Hungary	KP701905	KP701782	KP702028
*Cladosporioides*	*C*. *cladosporioides*	109-I4	October-09		S	Denmark	KP701920	KP701797	KP702043
*Cladosporioides*	*C*. *cladosporioides*	109-I6	October-09		S	Denmark	KP701922	KP701799	KP702045
*Cladosporioides*	*C*. *cladosporioides*	127-D8	April-09		A	The Netherlands	KP701933	KP701810	KP702055
*Cladosporioides*	*C*. *cladosporioides*	147-A9	Winter 2009		Indoor	Hungary	KP701941	KP701818	KP702063
*Cladosporioides*	*C*. *delicatulum*	082-F3	November-08	139574	A	Weert, The Netherlands	KP701880	KP701757	KP702003
*Cladosporioides*	*C*. *delicatulum*	134-D3	June-10		Indoor	Algeria	KP701939	KP701816	KP702061
*Cladosporioides*	*C*. *delicatulum*	145-C4	November-10		Indoor	Germany	KP701940	KP701817	KP702062
*Cladosporioides*	*C*. *delicatulum*	167-H5	May-11		A	Poland	KP701964	KP701841	KP702086
*Cladosporioides*	*C*. *globisporum*	220-D4	August-12	139587	S	Utrecht, The Netherlands	KP701967	KP701844	KP702089
*Cladosporioides*	*C*. *inversicolor*	072-C9	July-08	139573	A	Amsterdam, The Netherlands	KP701874	KP701751	KP701997
*Cladosporioides*	*C*. *inversicolor*	108-F8	September-09		Indoor	France	KP701908	KP701785	KP702031
*Cladosporioides*	*C*. *perangustum*	220-D5	August-12	139588	S	Utrecht, The Netherlands	KP701968	KP701845	KP702090
*Cladosporioides*	*C*. *perangustum*	127-E1	April-09		A	USA	KP701934	KP701811	KP702056
*Cladosporioides*	*C*. *pseudocladosporioides*	084-F1	December-08	139575	Indoor	Germany	KP701881	KP701758	KP702004
*Cladosporioides*	*C*. *pseudocladosporioides*	079-F4	October-08		S	s Hertogenbosch, The Netherlands	KP701877	KP701754	KP702000
*Cladosporioides*	*C*. *pseudocladosporioides*	150-C1	February-11		A	Portugal	KP701943	KP701820	KP702065
*Cladosporioides*	*C*. *pseudocladosporioides*	151-D1	February-11		A	Portugal	KP701946	KP701823	KP702068
*Cladosporioides*	*C*. *pseudocladosporioides*	151-G7	February-11		A	Portugal	KP701949	KP701826	KP702071
*Cladosporioides*	*C*. *subuliforme*	130-H8	May-10		A	Thailand	KP701938	KP701815	KP702060
*Cladosporioides*	*C*. *tenuissimum*	109-A1	October-09		A	Thailand	KP701910	KP701787	KP702033
*Cladosporioides*	*C*. *tenuissimum*	130-F6	May-10		A	Thailand	KP701937	KP701814	KP702059
*Cladosporioides*	Unidentified	056-H7	August-07		S	Utrecht, The Netherlands	KP701871	KP701748	KP701994
*Cladosporioides*	Unidentified	072-E4	July-08		A	Amsterdam, The Netherlands	KP701875	KP701752	KP701998
*Cladosporioides*	Unidentified	084-F2	December-08		Indoor	Germany	KP701882	KP701759	KP702005
*Cladosporioides*	Unidentified	086-B3	December-08		S	s Hertogenbosch, The Netherlands	KP701886	KP701763	KP702009
*Cladosporioides*	Unidentified	108-G8	October-09		A	Thailand	KP701909	KP701786	KP702032
*Cladosporioides*	Unidentified	109-E7	October-09		S	Denmark	KP701913	KP701790	KP702036
*Cladosporioides*	Unidentified	109-F2	October-09		S	Denmark	KP701915	KP701792	KP702038
*Herbarum*	*C*. *allicinum*	109-I5	October-09	139578	S	Denmark	KP701921	KP701798	KP702044
*Herbarum*	*C*. *allicinum*	121-H1	January-10	139580	Indoor	Germany	KP701930	KP701807	a
*Herbarum*	*C*. *allicinum*	073-C8	July-08		Indoor	Greece	KP701876	KP701753	KP701999
*Herbarum*	*C*. *allicinum*	084-F3	December-08		Indoor	Germany	KP701883	KP701760	KP702006
*Herbarum*	*C*. *allicinum*	086-D5	December-08		S	s Hertogenbosch, The Netherlands	KP701888	KP701765	KP702011
*Herbarum*	*C*. *allicinum*	089-B9	January-09		A	Rijssen, The Netherlands	KP701891	KP701768	KP702014
*Herbarum*	*C*. *allicinum*	089-G4	January-09		A	Eindhoven, The Netherlands	KP701894	KP701771	KP702017
*Herbarum*	*C*. *allicinum*	089-G6	January-09		A	Eindhoven, The Netherlands	KP701895	KP701772	KP702018
*Herbarum*	*C*. *allicinum*	089-H3	January-09		A	Eindhoven, The Netherlands	KP701896	KP701773	KP702019
*Herbarum*	*C*. *allicinum*	090-D3	January-09		S	Rijswijk, The Netherlands	KP701900	KP701777	KP702023
*Herbarum*	*C*. *allicinum*	101-A1	May-09		S	The Netherlands	KP701903	KP701780	KP702026
*Herbarum*	*C*. *allicinum*	106-C2	July-09		A	Amsterdam, The Netherlands	KP701906	KP701783	KP702029
*Herbarum*	*C*. *allicinum*	109-E6	October-09		S	Denmark	KP701912	KP701789	KP702035
*Herbarum*	*C*. *allicinum*	109-F3	October-09		S	Denmark	KP701916	KP701793	KP702039
*Herbarum*	*C*. *allicinum*	109-F5	October-09		S	Denmark	KP701918	KP701795	KP702041
*Herbarum*	*C*. *allicinum*	110-B7	October-09		A	Denmark	KP701923	KP701800	KP702046
*Herbarum*	*C*. *allicinum*	111-A5	October-09		A	Denmark	KP701924	KP701801	KP702047
*Herbarum*	*C*. *allicinum*	249-G3	March-13		S	The Hague, The Netherlands	KP701975	KP701852	KP702097
*Herbarum*	*C*. *ramotenellum*	089-C1	January-09	139577	A	Rijssen, The Netherlands	KP701892	KP701769	KP702015
*Herbarum*	*C*. *ramotenellum*	255-G5	March-13	139590	S	Utrecht, The Netherlands	KP701983	KP701860	KP702105
*Herbarum*	*C*. *ramotenellum*	109-F4	October-09		S	Denmark	KP701917	KP701794	KP702040
*Herbarum*	*C*. *ramotenellum*	151-G3	February-11		A	Portugal	KP701947	KP701824	KP702069
*Herbarum*	*C*. *ramotenellum*	151-G6	February-11		A	Portugal	KP701948	KP701825	KP702070
*Herbarum*	*C*. *ramotenellum*	152-D9	February-11		A	Portugal	KP701950	KP701827	KP702072
*Herbarum*	*C*. *ramotenellum*	249-F5	March-13		S	The Hague, The Netherlands	KP701972	KP701849	KP702094
*Herbarum*	*C*. *sinuosum*	109-I2	October-09		S	Denmark	KP701919	KP701796	KP702042
*Herbarum*	*C*. *tenellum*	127-D7	January-09	139582	A	USA	KP701932	KP701809	KP702054
*Herbarum*	Unidentified	090-H8	January-09		S	Utrecht, The Netherlands	KP701901	KP701778	KP702024
*Sphaerospermum*	*C*. *dominicanum*	255-H5	March-13	139591	S	Utrecht, The Netherlands	KP701987	KP701864	KP702109
*Sphaerospermum*	*C*. *dominicanum*	249-F4	March-13		S	The Hague, The Netherlands	KP701971	KP701848	KP702093
*Sphaerospermum*	*C*. *dominicanum*	255-F7	March-13		S	Utrecht, The Netherlands	KP701979	KP701856	KP702101
*Sphaerospermum*	*C*. *halotolerans*	147-B9	Winter 2009	139583	Indoor	Hungary	KP701942	KP701819	KP702064
*Sphaerospermum*	*C*. *halotolerans*	161-D3	May-11	139585	S	Gilze, The Netherlands	KP701955	KP701832	KP702077
*Sphaerospermum*	*C*. *halotolerans*	164-A6	August-11	139586	S	Veenendaal, The Netherlands	KP701963	KP701840	KP702085
*Sphaerospermum*	*C*. *halotolerans*	220-D7	August-12	139589	S	Utrecht, The Netherlands	KP701969	KP701846	KP702091
*Sphaerospermum*	*C*. *halotolerans*	049-E7	September-07		S	Utrecht, The Netherlands	KP701869	KP701746	KP701992
*Sphaerospermum*	*C*. *halotolerans*	102-A1	May-09		S	Hungary	KP701904	KP701781	KP702027
*Sphaerospermum*	*C*. *halotolerans*	109-D3	October-09		A	Thailand	KP701911	KP701788	KP702034
*Sphaerospermum*	*C*. *halotolerans*	114-H7	December-09		S	Thailand	KP701925	KP701802	KP702048
*Sphaerospermum*	*C*. *halotolerans*	114-I3	December-09		S	Thailand	KP701926	KP701803	KP702049
*Sphaerospermum*	*C*. *halotolerans*	117-H3	January-10		Indoor	The Netherlands	KP701929	KP701806	KP702052
*Sphaerospermum*	*C*. *halotolerans*	127-E8	April-09		A	USA	KP701936	KP701813	KP702058
*Sphaerospermum*	*C*. *halotolerans*	153-C3	February-11		S	Utrecht, The Netherlands	KP701952	KP701829	KP702074
*Sphaerospermum*	*C*. *halotolerans*	160-I2	March-11		S	Utrecht, The Netherlands	KP701953	KP701830	KP702075
*Sphaerospermum*	*C*. *halotolerans*	161-D5	May-11		S	Gilze, The Netherlands	KP701957	KP701834	KP702079
*Sphaerospermum*	*C*. *halotolerans*	161-D6	May-11		S	Gilze, The Netherlands	KP701958	KP701835	KP702080
*Sphaerospermum*	*C*. *halotolerans*	220-D3	August-12		S	Utrecht, The Netherlands	KP701966	KP701843	KP702088
*Sphaerospermum*	*C*. *halotolerans*	249-F9	March-13		S	The Hague, The Netherlands	KP701974	KP701851	KP702096
*Sphaerospermum*	*C*. *halotolerans*	249-G4	March-13		S	The Hague, The Netherlands	KP701976	KP701853	KP702098
*Sphaerospermum*	*C*. *halotolerans*	255-F8	March-13		S	Utrecht, The Netherlands	KP701980	KP701857	KP702102
*Sphaerospermum*	*C*. *halotolerans*	255-G4	March-13		S	Utrecht, The Netherlands	KP701982	KP701859	KP702104
*Sphaerospermum*	*C*. *halotolerans*	255-G6	March-13		S	Utrecht, The Netherlands	KP701984	KP701861	KP702106
*Sphaerospermum*	*C*. *halotolerans*	255-H3	March-13		S	Utrecht, The Netherlands	KP701985	KP701862	KP702107
*Sphaerospermum*	*C*. *halotolerans*	257-F4	March-13		S	Utrecht, The Netherlands	KP701989	KP701866	KP702111
*Sphaerospermum*	*C*. *langeronii*	124-D5	February-10	139581	A	Ospel, The Netherlands	KP701931	KP701808	KP702053
*Sphaerospermum*	*C*. *langeronii*	085-H6	December-08		A	s Hertogenbosch, The Netherlands	KP701885	KP701762	KP702008
*Sphaerospermum*	*C*. *sphaerospermum*	084-F4	December-08	139576	Indoor	Germany	KP701884	KP701761	KP702007
*Sphaerospermum*	*C*. *sphaerospermum*	117-G5	January-10	139579	Indoor	The Netherlands	KP701927	KP701804	KP702050
*Sphaerospermum*	*C*. *sphaerospermum*	150-H8	February-11	139584	A	Portugal	KP701944	KP701821	KP702066
*Sphaerospermum*	*C*. *sphaerospermum*	017-C7	May-06		S	Eindhoven, The Netherlands	KP701867	KP701744	KP701990
*Sphaerospermum*	*C*. *sphaerospermum*	049-H5	September-07		S	Utrecht, The Netherlands	KP701870	KP701747	KP701993
*Sphaerospermum*	*C*. *sphaerospermum*	086-E7	December-08		Indoor	Utrecht, The Netherlands	KP701889	KP701766	KP702012
*Sphaerospermum*	*C*. *sphaerospermum*	086-E8	December-08		Indoor	Utrecht, The Netherlands	KP701890	KP701767	KP702013
*Sphaerospermum*	*C*. *sphaerospermum*	089-E9	January-09		A	Eindhoven, The Netherlands	KP701893	KP701770	KP702016
*Sphaerospermum*	*C*. *sphaerospermum*	090-A1	January-09		A	Eindhoven, The Netherlands	KP701897	KP701774	KP702020
*Sphaerospermum*	*C*. *sphaerospermum*	090-I1	January-09		S	Utrecht, The Netherlands	KP701902	KP701779	KP702025
*Sphaerospermum*	*C*. *sphaerospermum*	106-D4	July-09		A	Amsterdam, The Netherlands	KP701907	KP701784	KP702030
*Sphaerospermum*	*C*. *sphaerospermum*	117-H2	January-10		Indoor	The Netherlands	KP701928	KP701805	KP702051
*Sphaerospermum*	*C*. *sphaerospermum*	150-I8	February-11		A	Portugal	KP701945	KP701822	KP702067
*Sphaerospermum*	*C*. *sphaerospermum*	153-B7	February-11		S	Utrecht, The Netherlands	KP701951	KP701828	KP702073
*Sphaerospermum*	*C*. *sphaerospermum*	160-I4	March-11		S	Utrecht, The Netherlands	KP701954	KP701831	KP702076
*Sphaerospermum*	*C*. *sphaerospermum*	161-D4	May-11		S	Gilze, The Netherlands	KP701956	KP701833	KP702078
*Sphaerospermum*	*C*. *sphaerospermum*	161-D7	May-11		S	Gilze, The Netherlands	KP701959	KP701836	KP702081
*Sphaerospermum*	*C*. *sphaerospermum*	161-D8	May-11		S	Gilze, The Netherlands	KP701960	KP701837	KP702082
*Sphaerospermum*	*C*. *sphaerospermum*	161-D9	May-11		S	Gilze, The Netherlands	KP701961	KP701838	KP702083
*Sphaerospermum*	*C*. *sphaerospermum*	194-A4	April-12		S	Utrecht, The Netherlands	KP701965	KP701842	KP702087
*Sphaerospermum*	*C*. *sphaerospermum*	244-C6			S	Germany	KP701970	KP701847	KP702092
*Sphaerospermum*	*C*. *sphaerospermum*	249-F7	March-13		S	The Hague, The Netherlands	KP701973	KP701850	KP702095
*Sphaerospermum*	*C*. *sphaerospermum*	249-G5	March-13		S	The Hague, The Netherlands	KP701977	KP701854	KP702099
*Sphaerospermum*	*C*. *sphaerospermum*	255-G3	March-13		S	Utrecht, The Netherlands	KP701981	KP701858	KP702103
*Sphaerospermum*	*C*. *sphaerospermum*	255-H4	March-13		S	Utrecht, The Netherlands	KP701986	KP701863	KP702108
*Sphaerospermum*	*C*. *sphaerospermum*	255-H7	March-13		S	Utrecht, The Netherlands	KP701988	KP701865	KP702110
*Sphaerospermum*	Unidentified	162-A4	June-11		S	Arnhem, The Netherlands	KP701962	KP701839	KP702084

Strains with a CBS number are deposited in the CBS strain collection. A and S represent air and swab samples, respectively, and samples taken from unknown indoor sources are indicated with “Indoor”. The GenBank numbers of the ITS, *TEF* and *ACT* sequences are shown in the last three columns. Strains used for a_w_ experiments are underlined. ^a^DTO 121-H1 is identified using the sequences of ITS and *TEF* only.

### Growth on media with lowered water activity

Water activity (a_w_) of the medium was set between 0.99 and 0.75 by substitution of water with 0–50% glycerol (v/v). A volume of 23 ml of medium was poured in Petri dishes with vents (Greiner, Bio-One B. V., Alphen aan de Rijn, The Netherlands) and left to solidify for 24 h on the bench with the lid closed. The a_w_ of control (non-inoculated) plates of the different glycerol-agar mixtures was determined before and after growth experiments using a Novasina labmaster-a_w_ (Novasina, Lachen, Switzerland) and samples with a diameter of 5 cm. The a_w_ changed only marginally during 3 weeks of storage (ranging between a decrease of 0.01 a_w_ unit in 5% glycerol plates and an increase of 0.03 units in 50% glycerol plates).

Agar plates were inoculated with 3 μl spore solution containing 1 x 10^6^ spores ml ^-1^ harvested from 7-days-old MEA-grown cultures. Conidia were collected in 10 mM ACES, 0.02% Tween 80. The spore suspension was filtered over sterile glass wool to remove hyphal fragments. Spores were counted using a Bürker-Türk haemocytometer and the suspension was diluted to 1 x 10^6^ spores ml ^-1^.

### DNA sequencing and molecular analysis

Genomic DNA was isolated from 7-days-old cultures using the Ultraclean Microbial DNA isolation kit (MoBio Laboratories, USA) according to the manufacturer’s instructions. ITS, *ACT*, and *TEF* loci were amplified, using the primers shown in Table 2 [28–31], as described earlier in Houbraken and Samson (2011) [32]. The fragments were sequenced with the BigDye Terminator v3.1 Cycle Sequencing Kit (Applied Biosystems, USA). The products were analyzed on an ABI Prism 3730 XL DNA Sequencer (Applied Biosystems, USA). Sequences were assembled by using the forward and reverse sequences with the program SeqMan from the LaserGene 9 package (DNAstar, USA). The ITS, *ACT*, and *TEF* sequences were concatenated resulting in 1132 nucleotide long sequences. They were aligned to sequences of 70 taxa described by Bench et al. (2012) [25] using the online version of MAFFT [33]. The resulting alignments were manually improved and phylogenetic analyses were conducted using MEGA version 5 software [34]. A phylogenetic tree (S1 Fig) was made using pairwise deletion neighbor joining with 1000 bootstrap repetitions and the nucleotide substitution model, Tamura Nei, with gamma distribution parameter 4.

**Table 2:** Primers used for PCR and sequencing.

Primer:	Locus:	Primer sequence:	Reference:
V9G	Internal Transcribed Spacer	TTACGTCCCTGCCCTTTGTA	[29, 31]
LS266	Internal Transcribed Spacer	GCATTCCCAAACAACTCGACTC	[29, 31]
ACT-512F	Actin	ATGTGCAAGGCCGGTTTCGC	[28]
ACT-783R	Actin	TACGAGTCCTTCTGGCCCAT	[28]
EF1-728F	Translation Elongation factor 1 alpha	CATCGAGAAGTTCGAGAAGG	[28]
EF2	Translation Elongation factor 1 alpha	GGARGTACCAGTSATCATGTT	[30]

### Statistics

A Pearson’s Chi-square test was performed using Microsoft Office Professional Plus Excel 2010.

## Results

### Identification of *Cladosporium* species of indoor origin

**Fig 1:**
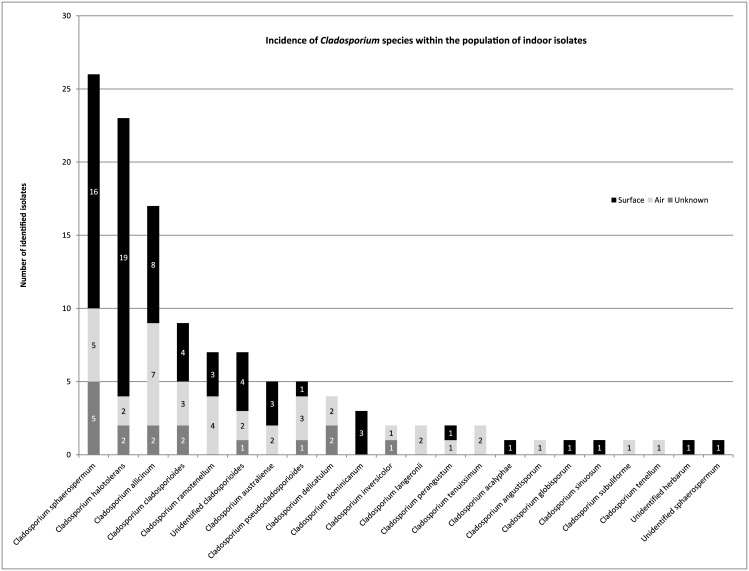
Incidence of *Cladosporium* species within the population of indoor isolates. The origin of the samples (air, indoor surface or unknown origin) is also shown.

The *Cladosporium* collection of the Applied and Industrial Mycology group of the CBS Fungal Biodiversity Centre includes 67 strains from indoor surfaces (swab samples), 39 strains from air samples, and 17 isolates from an unknown indoor origin (Table 1). Out of the 123 indoor *Cladosporium* isolates, 74 originate from the Netherlands, and 49 from Denmark, France, Germany, Greece, Hungary, Poland, Portugal, Thailand, Algeria and The United States of America. The 123 isolates were identified based on the ITS, *ACT* and *TEF* sequences and a phylogenetic tree was constructed (S1 Fig). The collection consisted of 55, 41 and 27 isolates belonging to the species complexes of *C*. *sphaerospermum*, *C*. *cladosporioides* and *C*. *herbarum*, respectively (Table 3). Nine isolates could not be identified to species level since the sequences did not align well with any of the type species used for comparison and might represent new species. From these species, seven grouped in the *C*. *cladosporioides* species complex, and one in the *C*. *herbarum* and one in the *C*. *sphaerospermum* species complex. A total number of 19 different species were identified, of which *C*. *sphaerospermum* was most common, followed by *C*. *halotolerans* and *Cladosporium allicinum* (Fig 1). To compare the species distribution of swab and air samples, the 17 strains of unknown indoor origin were excluded. The *C*. *sphaerospermum* species complex made up more than 44.7% of the total number of 106 isolates and even 58.2% of the swab isolates (Table 3). The *C*. *cladosporioides* species complex comprised 33.3% of the indoor isolates and 22.4% of the swab isolates. The *C*. *herbarum* species complex comprised 22.0% of the indoor isolates and 19.4% of the swab isolates (Table 3). A Pearson’s Chi-square test shows significant increased numbers (p-value ≤ 0.05) of *C*. *sphaerospermum* species complex in swab samples compared to air samples. This was not observed in the case of the *C*. *herbarum* and *C*. *cladosporioides* species complexes.

**Table 3:** The number of isolates from indoor air and surface samples from the 3 species complexes.

Species complex	Surface	%	Air	%	Unknown	%	Total	%
***C*. *sphaerospermum***	39	58.2%	9	23.1%	7	41.2%	55	44.7%
***C*. *cladosporioides***	15	22.4%	18	46.2%	8	47.0%	41	33.3%
***C*. *herbarum***	13	19.4%	12	30.8%	2	11.8%	27	22.0%
**Total**	67	100%	39	100%	17	100%	123	100%

### Growth of indoor *Cladosporium* species at lower water activity

**Fig 2:**
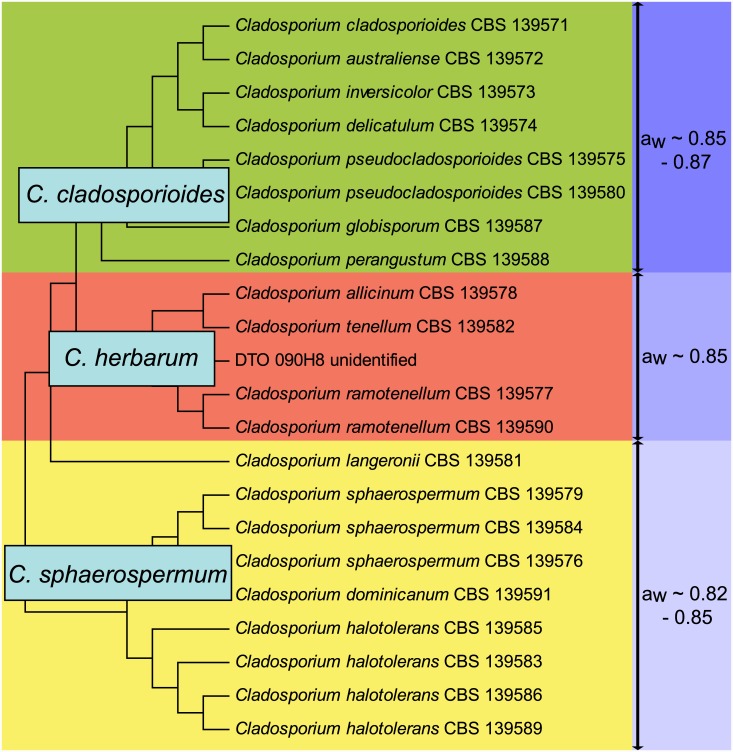
Schematic dendrogram showing the minimal water activity needed for growth of 22 selected indoor *Cladosporium* species. Green, red and yellow represent the *C*. *cladosporioides*, *C*. *herbarum* and *C*. *sphaerospermum* species complexes, respectively.

**Fig 3:**
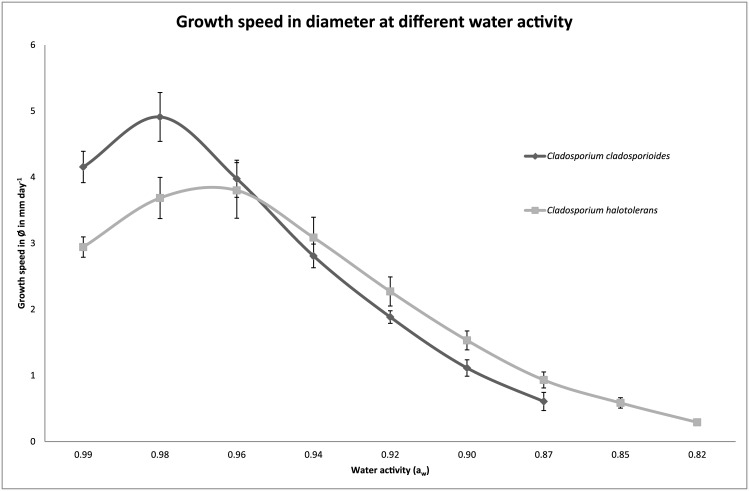
Increase in colony diameter in mm day^-1^ of *Cladosporium cladosporioides* CBS 139571 and *Cladosporium halotolerans* CBS 139586 at varying a_w_ at 25°C.

Growth of 22 indoor isolates of *Cladosporium* was compared on media with water activity (a_w_) values ranging from 0.75 to 0.99. These isolates include 9, 8 and 5 isolates of the *C*. *sphaerospermum*, *C*. *cladosporioides* and *C*. *herbarum* species complexes, respectively, which represent 14 different species (Fig 2). Moreover, the selection made up 7 air samples, 9 swab samples and 6 indoor samples of which the origin (air or swab) is not known. The minimal a_w_ needed to enable growth during a 3-week-period, ranged from 0.82 to 0.87 for the selected *Cladosporium* species (Table 4). The air isolates showed a minimal a_w_ of 0.82 to 0.87, while the swab samples showed a minimal a_w_ of 0.82 to 0.85. All isolates of *C*. *sphaerospermum* and *C*. *halotolerans* grew at a_w_ ≥ 0.82. Interestingly, these are also the two most abundant species in indoor air and swab samples. *Cladosporium dominicanum* and *Cladosporium langeronii* that also belong to the *C*. *sphaerospermum* species complex grew at a_w_ ≥ 0.85. All selected species from the *C*. *herbarum* species complex (i.e. *C*. *allicinum*, *Cladosporium ramotenellum* and *Cladosporium tenellum* and an unidentified species) also grew at a_w_ ≥ 0.85. *Cladosporium globisporum* and *Cladosporium perangustum* of the *C*. *cladosporioides* species complex grew at a_w_ ≥ 0.85, while *Cladosporium australiense*, *Cladosporium cladosporioides*, *Cladosporium delicatulum* and *Cladosporium inversicolor* only grew at a_w_ ≥ 0.87. The ability to grow at a particular water activity even differed within a species. *Cladosporium pseudocladosporioides* CBS 139575 could grow at a_w_ of ≥ 0.85, while *C*. *pseudocladosporioides* CBS 139580 grew at a_w_ of ≥ 0.87 (Table 4). Fig 2 summarizes the data and shows that the isolates from the *C*. *sphaerospermum* species complex, that are most abundant on indoor surfaces, grow at the lowest water activity. Notably, growth of *C*. *cladosporioides* at water activity of 0.98 is clearly faster compared to that of *C*. *halotolerans* (Fig 3 and S2 Fig). This shows that the modest xerophily of the *C*. *sphaerospermum* species complex is not the result of its higher growth rates compared to *C*. *cladosporioides* and *C*. *herbarum*.

**Table 4:** Growth of 22 isolates of *Cladosporium* on MEA with different water activity.

Species	Strain nr.	Growth rate in increase of Ø (mm day -1) at a_w_ opt	a_w_^opt^	a_w_^min^
*C*. *allicinum*	139578	4.40	0.98	0.85
*C*. *australiense*	139572	4.84	0.98	0.87
*C*. *cladosporioides*	139571	4.91	0.98	0.87
*C*. *delicatulum*	139574	1.98	0.98	0.87
*C*. *dominicanum*	139591	1.67	0.94	0.85
*C*. *globisporum*	139587	4.24	0.98	0.85
*C*. *halotolerans*	139583	3.98	0.98	0.82
*C*. *halotolerans*	139585	3.13	0.96	0.82
*C*. *halotolerans*	139586	3.80	0.96	0.82
*C*. *halotolerans*	139589	3.91	0.96	0.82
*C*. *inversicolor*	139573	3.51	0.98	0.87
*C*. *langeronii*	139581	1.07	0.94	0.85
*C*. *perangustum*	139588	4.35	0.98	0.85
*C*. *pseudocladosporioides*	139575	5.66	0.98	0.85
*C*. *pseudocladosporioides*	139580	5.53	0.98	0.87
*C*. *ramotenellum*	139577	4.16	0.98	0.85
*C*. *ramotenellum*	139590	4.40	0.98	0.85
*C*. *sphaerospermum*	139576	4.47	0.96	0.82
*C*. *sphaerospermum*	139579	3.72	0.96	0.82
*C*. *sphaerospermum*	139584	3.45	0.96	0.82
*C*. *tenellum*	139582	3.55	0.98	0.85
Unidentified *herbarum*	DTO 090-H8	2.83	0.96	0.85

Growth rate (increase in diameter, Ø) in mm day^-1^ is shown for each isolate at the optimal a_w_ at 25°C.

## Discussion

*Cladosporium* species are among the most abundant fungi in outdoor and indoor air [8, 11, 13]. In fact, *C*. *cladosporioides* was reported to be the most predominant fungus in houses in Ontario and Atlanta [11, 13]. Recently, classification of the genus *Cladosporium* has been revised on the basis of morphology and multi-locus sequencing [19, 20, 25, 26]. This novel classification was used in our study where we sequenced the same genes and compared the data. Our data showed that members of the *C*. *sphaerospermum* species complex are the most predominant indoor fungi, which were found in 44.7% of the indoor samples and 58% of indoor swabs. Notably, moderate xerotolerancy of the species (0.82–0.85) correlated with the presence of these species on indoor surfaces.

Generally, indoor levels of fungi are lower than outdoor and are related to the outdoor species composition. The composition of fungi in the indoor environment is highly similar to the outdoor air in well-ventilated houses [6, 8, 9, 13, 35, 36]. Indoor surfaces have been described as passive collectors of airborne fungi of outdoor origin. Yet, different surface types harbor different fungal populations. This indicates that the composition of fungal populations on surfaces can deviate from that of the outdoor and indoor air. This is supported by our finding that *C*. *sphaerospermum* species complex isolates are more prominent on indoor surfaces when compared to air samples. These effects might be more pronounced in winter when air replacement with outdoor air in dwellings is markedly lower [8].

*Cladosporium* species are not reported as producers of mycotoxins. Nonetheless, they may represent a threat to health. *Cladosporium* species from all three species complexes are reported to cause fungal allergies [4, 37–41], especially in patients with severe asthma [3, 42]. The dominance of the *C*. *sphaerospermum* species complex in indoor environments is of interest as *C*. *herbarum* is the most studied species in allergy research [43, 44]. Cell extracts of *C*. *herbarum* are used for allergy tests, particularly in skin prick tests [37, 44]. Not much is known about the *Cladosporium* proteins raising allergy. Enolase, aldehyde dehydrogenase and mannitol dehydrogenase (*MDH*) are known allergens from *C*. *herbarum* [43]. The *C*. *herbarum MDH* shows an identity of 83.9% with the *Cladosporium fulvum MDH* [45] and 92.6% identity with the same locus in an isolate of *C*. *sphaerospermum* isolated from blood culture and of which the full genome is sequenced [46]. However, we would like to stress that the identification of *C*. *sphaerospermum* in the abovementioned study is based on the sequence of the ITS region. By using BLAST we have also identified the sequences of the *TEF*, ITS and *ACT* loci. These sequences were aligned to data of Bensch *et*. *al*. and group in the phylogenetic tree with *C*. *halotolerans* as is shown in S1 Fig. [25]. Therefore, we propose to evaluate cross-reactivity of the allergens of the different *Cladosporia*. Specifically the common indoor fungi, *C*. *sphaerospermum*, *C*. *halotolerans* and *C*. *allicinum*, should be evaluated to assess whether the screening panels of these fungi have to be adapted.

## Supporting Information

S1 Fig Phylogram based on neighbor joining analysis showing all used isolates, including *C*. *sphaerospermum* UM843, supplemented with reference sequences from Bensch et al. (2012). Statistical support was calculated by using 1000 bootstrap replicates. Bootstrap values below 70% are not shown. This phylogram was made using pairwise deletion and the nucleotide substitution model, Tamura Nei, with gamma distribution parameter 4.
(EPS)

S2 Fig The growth speed (increase of colony diameter in mm day^-1^) of 22 *Cladosporium* species at different a_w_ at 25°C. (EPS)
